# The Neuroepithelium Disruption Could Generate Autoantibodies against AQP4 and Cause Neuromyelitis Optica and Hydrocephalus

**DOI:** 10.1155/2014/580572

**Published:** 2014-10-29

**Authors:** Leandro Castañeyra-Ruiz, Ibrahim González-Marrero, Agustín Castañeyra-Ruiz, Juan M. González-Toledo, María Castañeyra-Ruiz, Francisco J. Perez-Molto, Emilia M. Carmona-Calero, Agustín Castañeyra-Perdomo

**Affiliations:** ^1^Departamento de Anatomía, Facultad de Medicina, Universidad de La Laguna, 38200 La Laguna, Tenerife, Islas Canarias, Spain; ^2^Instituto de Investigación y Ciencias de Puerto del Rosario, 35600 Puerto del Rosario, Fuerteventura, Islas Canarias, Spain; ^3^Departamento de Ciencias Morfológicas, Universidad de Valencia, 46010 Valencia, Spain

## Abstract

Neuromyelitis optica is an inflammatory disease characterized by neuritis and myelitis of the optic nerve. Its physiopathology is connected with the aquaporin-4 water channel, since antibodies against aquaporin-4 have been found in the cerebrospinal fluid and blood of neuromyelitis optica patients. The seropositivity for aquaporin-4 antibodies is used for the diagnosis of neuromyelitis optica or neuromyelitis optica spectrum disease. On the other hand, aquaporin-4 is expressed in astrocyte feet in the brain-blood barrier and subventricular zones of the brain ventricles. Aquaporin-4 expression is high in cerebrospinal fluid in hydrocephalus. Furthermore, neuroepithelial denudation precedes noncommunicating hydrocephalus and this neuroepithelial disruption could allow aquaporin-4 to reach anomalous brain areas where it is unrecognized and induce the generation of aquaporin-4 antibodies which could cause the neuromyelitis optica and certain types of hydrocephalus.

## 1. Introduction

Neuromyelitis optica (NMO) or Devic's disease is an autoimmune disorder affecting the optic nerve and spinal cord, where the aquaporin-4 water channel (AQP4) plays an important role. Thus, antibodies against AQP4 are detectable in the cerebrospinal fluid (CSF) of most patients with neuromyelitis optica (NMO) spectrum disease, mainly when it is worsening. Currently, seropositivity for AQP4-Ab is the only validated diagnostic biomarker for autoimmune optic neuropathies [1] since the diagnostic specificity of this test is almost 99%. However, there is about a 5% overlap with other autoimmune diseases [2]. These findings support the suggestion that any patient with an autoimmune optic neuropathy which is seropositive for AQP4 autoantibodies should be given a diagnosis of NMO or NMO spectrum disease (NMOSD) [3, 4].

However, AQP4 presence in the CSF has also been proposed as a diagnostic biomarker of hydrocephalus type [5]; additionally the sites and mechanisms of CSF reabsorption are relevant to the pathophysiology of hydrocephalus [6]. It has traditionally been supposed that CSF reabsorption mainly occurred via arachnoid granulations, but increasing evidence points towards other drainage sites. Studies [7, 8] indicate the existence of a brain-wide pathway facilitating the exchange of CSF and interstitial fluid via para-arterial CSF influx, paravenous interstitial clearance, and a transparenchymal pathway dependent on water transport through astrocytic AQP4 channels.

## 2. Aquaporin-4 and Hydrocephalus

**Figure 1:**
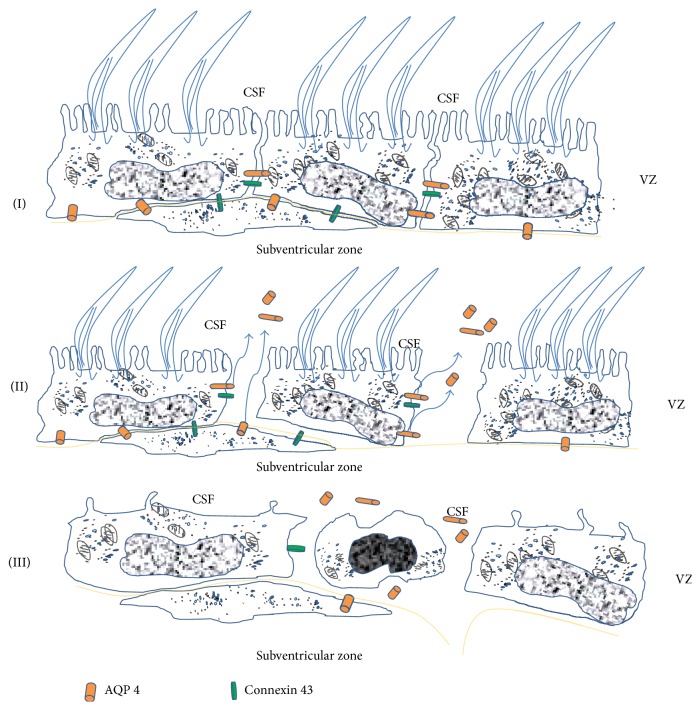
Drawing of ventricular zone disruption in hydrocephalus. AQP4 leaking into the CSF. Modified from Castaneyra-Ruiz et al. 2014 and Rodriguez et al. 2012 [16, 27]. CSF = cerebroespinal fluid, VZ = ventricular zone.

**Figure 2:**
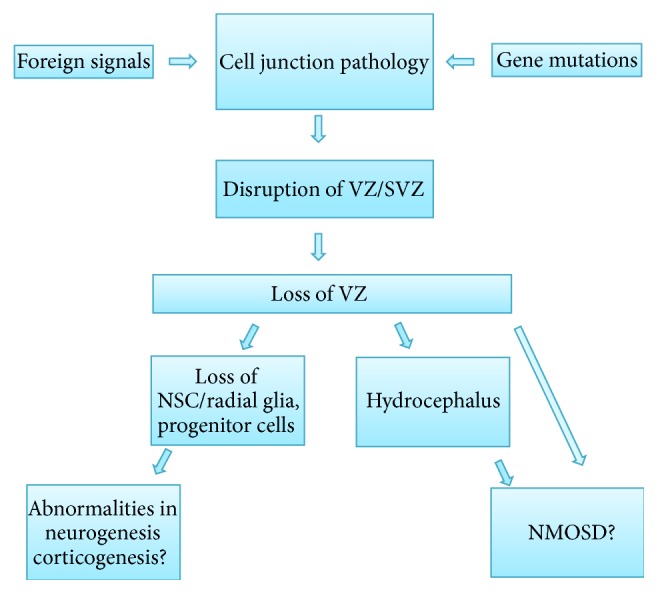
Flow chart representing the hypothesis that the cell junction pathology of the VZ cells leads to hydrocephalus, abnormal neurogenesis, and certain types of NMO. Modified from Rodriguez et al. 2012 [16]. NMO = neuromyelitis optica, NMOSD = neuromyelitis optica spectrum disease, NSC = neural stem cells, SVZ = subventricular zone, and VZ = ventricular zone.

Aquaporin-4 (AQP4) is a water channel mainly located at the end feet of astrocytes on the ependymal layer and in the blood-brain barrier (BBB) and is associated with the elimination of cerebral edema via this route [9]. AQP4 levels are significantly altered in kaolin-induced hydrocephalus, suggesting that AQP4 could play an important neurodefensive role in hydrocephalus and CSF disorders [10, 11]. An increase in CSF AQP4 in hydrocephalus is reported in a previous work [5] and this may occur as a consequence of the loss of communication between ependyma and subsequent cell disruption; thus AQP4 would pass into the CSF [5]. This ependymal loss appears much earlier than the increase of intracranial pressure and the ventriculomegaly and is therefore the first recognizable event in hydrocephalus pathology, and this is accompanied by a microglial and astroglial cell reaction; the subependymal astroglial cells respond by proliferation in such a way that they form a glial scar-like covering of the ventricular surface to replace the ependymal epithelium [12, 13]. At the same time, if cellular death or disruption occurs, the AQP4 could be in contact with the ventricular lumen and may pass into the CSF. Furthermore, the occurrence of various stages of ependymal denudation within full-term spina bifida aperta (SBA) fetuses suggests that there may be a continuation of the process after birth and that cases of communicating hydrocephalus could soon develop into noncommunicating hydrocephalus (Figures 1 and 2) [8, 14–16]. Therefore, the increase of CSF AQP4 may be a consequence of ependymal disruption and the level of AQP4 in the CSF could be an indicator of the ependyma status and the stage of hydrocephalus [5]. Thus, an AQP4-dependent mechanism could facilitate reabsorption of CSF and clearance from the parenchyma into the microvasculature.

## 3. Aquaporin-4 and Neuromyelitis Optica

Aquaporin-4 is located in optic nerve and other ocular tissues (Table 1) [17, 18]. In a state of subacutely acquired AQP4 dysfunction, as occurs in NMO, altered CSF reabsorption could further exacerbate hydrocephalus through a nonobstructive mechanism [19]. Furthermore, the 1% frequency of obstructive hydrocephalus is observed in patients with NMOSD, which is far greater than in the general adult population. The incidence of all types of hydrocephalus, annual numbers of new ventricular shunts recorded in the Nationwide Inpatient Sample database and the Californian population, is 2.95 and 5.5 per 100,000, respectively [20–22]. Only 16.6% were for obstructive/noncommunicating hydrocephalus [20]. Hydrocephalus is related to NMO AQP4 seropositive patients in only one report by Clardy et al. [19] and in a poster presented in one neuroophthalmology meeting [23]. The cerebral aqueduct, an anatomically restricted access of the ventricular system, is lined with ependymal cells expressing AQP4 and inflammatory sequelae of IgG binding to AQP4 and it is thought that this is the region where scarring, occlusion, stenosis, or reduced compliance of the aqueductal channel leading to obstruction occurs [19].

NMO is an inflammatory disease of the central nervous system clinically characterized by a neuritis and transverse myelitis of the optic nerve. Likewise, the NMO has recently been defined as a disabling and autoimmune disease with an astrocyte pathology characterized by severe and recurrent attacks of optic neuritis and longitudinally extensive myelitis [24]. NMO was initially considered as an acute aggressive variant of multiple sclerosis (MS). However, the finding of autoantibodies against AQP4 in both the serum and CSF completely disassociated NMO from MS. Interestingly, AQP4-Ab were found in other pathologies connected to NMO which explains why these pathologies are now reclassified as NMOSD. The mechanisms underlying the pathophysiology of NMOSD as the production of autoantibodies against AQP4 are still unknown today. Normally, the sample used to test the presence of AQP4-Ab is blood serum, but the presence of AQP4-Ab in the CSF and not in the serum has been reported in some cases of NMO [25]; therefore, it could be clinically useful to test for the presence of CSF AQP4-Ab to consider a diagnosis of seronegative NMOSD. Other authors report that the AQP4-Ab come from the blood and pass into the CSF by a malfunction of the blood-CSF barrier [6], but how could that explain, as reported [24], the absence of AQP4-Ab in serum and their presence in the CSF? Given the wide range of illnesses covered by NMOSD and the anomalous presence of AQP4 described in the CSF in cases of hydrocephalus, bacterial meningitis, and diseases with inflammatory phenomena of the central nervous system [5, 26], the presence of AQP4 in the CSF is probably due to ependymal disruption phenomena. The possibility that there were neuroepithelium alterations in some cases of NMO, which allowed the anomalous presence of AQP4 in the CSF thereby causing an autoimmune reaction and the possible generation of AQP4-Ab, could explain the physiopathology of several kinds of NMOSD such as seronegative cases for AQP4-Ab [25].

## 4. Conclusion

The frequency of patients with NMOSD and obstructive hydrocephalus is greater than that in the general adult population [19]. One could therefore propose that a reason for this greater frequency is because neuroepithelial disruption could occur more slowly during the onset stage in some types of hydrocephalus; thus the anomalous presence of AQP4 in the CSF would be longer and the parenchymal-CSF barrier disruption (transparenchymal pathway) during this time would allow more brain AQP4 to be in contact with lymphocytes in encephalic regions where this does not normally occur and where brain AQP4 is unrecognized and consequently autoantibodies (AQP4-Ab) might be generated which would then attack the AQP4 surrounding the optic nerve, in the ciliary body and on the ventricular surface of the sylvian aqueduct, and thereby trigger NMO and noncommunicating hydrocephalus.

## Figures and Tables

**Table 1:** Expression of aquaporins in ocular tissue (Patil et al. 1997 and Tran et al. 2014) [17, 18].

Ocular tissue	AQP1	AQP3	AQP4	AQP5	AQP7	AQP9

Cornea	D	D	ND	D	D	ND
Iris	D	ND	D	D		ND
Lens	D	ND	D	D	D	ND
Trabecular meshwork	D	ND	ND	ND	D	ND
Ciliary	D	ND	D	ND	D	D
Retina	D	ND	D	ND	D	D
Choroid	D	ND	ND	ND	ND	
Optic nerve	ND	ND	D	ND	ND	D

AQP = aquaporin, D = detected, and ND = not detected.

## References

[1] Lennon P. V. A., Wingerchuk D. M., Kryzer T. J. (2004). A serum autoantibody marker of neuromyelitis optica: distinction from multiple sclerosis. *The Lancet*.

[2] Jarius S., Ruprecht K., Wildemann B. (2012). Contrasting disease patterns in seropositive and seronegative neuromyelitis optica: a multicentre study of 175 patients. *Journal of Neuroinflammation*.

[3] Petzold A., Plant G. T. (2014). Chronic relapsing inflammatory optic neuropathy: a systematic review of 122 cases reported. *Journal of Neurology*.

[4] Petzold A., Plant G. T. (2014). Diagnosis and classification of autoimmune optic neuropathy. *Autoimmunity Reviews*.

[5] Castañeyra-Ruiz L., González-Marrero I., González-Toledo J. M. (2013). Aquaporin-4 expression in the cerebrospinal fluid in congenital human hydrocephalus. *Fluids and Barriers of the CNS*.

[6] Jarius S., Franciotta D., Paul F. (2010). Cerebrospinal fluid antibodies to aquaporin-4 in neuromyelitis optica and related disorders: frequency, origin, and diagnostic relevance. *Journal of Neuroinflammation*.

[7] Iliff J. J., Wang M., Liao Y. (2012). A paravascular pathway facilitates CSF flow through the brain parenchyma and the clearance of interstitial solutes, including amyloid *β*. *Science Translational Medicine*.

[8] Castaneyra-Ruiz L., Gonzalez-Marrero I., Gonzalez-Toledo J. M. (2012). La Hidrocefalia Congénita. Consideraciones sobre las vías menores de producción y reabsorción del líquido cefalorraquídeo. *Majorensis*.

[9] Filippidis A. S., Kalani M. Y., Rekate H. L. (2012). Hydrocephalus and aquaporins: the role of aquaporin-4. *Hydrocephalus*.

[10] Skjolding A. D., Holst A. V., Broholm H., Laursen H., Juhler M. (2013). Differences in distribution and regulation of astrocytic aquaporin-4 in human and rat hydrocephalic brain. *Neuropathology and Applied Neurobiology*.

[11] Skjolding A. D., Rowland I. J., Søgaard L. V., Praetorius J., Penkowa M., Juhler M. (2010). Hydrocephalus induces dynamic spatiotemporal regulation of aquaporin-4 expression in the rat brain. *Cerebrospinal Fluid Research*.

[12] Páez P., Bátiz L.-F., Roales-Buján R. (2007). Patterned neuropathologic events occurring in hyh congenital hydrocephalic mutant mice. *Journal of Neuropathology and Experimental Neurology*.

[13] Roales-Buján R., Páez P., Guerra M. (2012). Astrocytes acquire morphological and functional characteristics of ependymal cells following disruption of ependyma in hydrocephalus. *Acta Neuropathologica*.

[14] Sival D. A., Guerra M., Den Dunnen W. F. A. (2011). Neuroependymal denudation is in progress in full-term human foetal spina bifida aperta. *Brain Pathology*.

[15] Wagner C., Batiz L. F., Rodríguez S. (2003). Cellular mechanisms involved in the stenosis and obliteration of the cerebral aqueduct of hyh mutant mice developing congenital hydrocephalus. *Journal of Neuropathology and Experimental Neurology*.

[16] Rodriguez E. M., Guerra M. M., Vio K. (2012). A cell junction pathology of neural stem cells leads to abnormal neurogenesis and hydrocephalus. *Bilogical Research*.

[17] Patil R. V., Saito I., Yang X., Wax M. B. (1997). Expression of aquaporins in the rat ocular tissue. *Experimental Eye Research*.

[18] Tran T. L., Bek T., la Cour M. (2014). Altered aquaporin expression in glaucoma eyes. *Acta Pathologica, Microbiologica, et Immunologica Scandinavica*.

[19] Clardy S. L., Lucchinetti C. F., Krecke K. N. (2014). Hydrocephalus in neuromyelitis optica. *Neurology*.

[20] Patwardhan R. V., Nanda A. (2005). Implanted ventricular shunts in the United States: the billion-dollar-a- year cost of hydrocephalus treatment. *Neurosurgery*.

[21] Wu Y., Green N. L., Wrensch M. R., Zhao S., Gupta N. (2007). Ventriculoperitoneal shunt complications in California: 1990 to 2000. *Neurosurgery*.

[22] Marrie R. A., Gryba C. (2013). The incidence and prevalence of neuromyelitis optica: a systematic review. *International Journal of Multiple Sclerosis Care*.

[23] Gratton S., Mora C. Unexplained hydrocephalus in a patient with neuromyelitis optica.

[24] Lucchinetti C. F., Guo Y., Popescu B. F. G., Fujihara K., Itoyama Y., Misu T. (2014). The pathology of an autoimmune astrocytopathy: Lessons learned from neuromyelitis optica. *Brain Pathology*.

[25] Klawiter E. C., Alvarez E., Xu J. (2009). NMO-IgG detected in CSF in seronegative neuromyelitis optica. *Neurology*.

[26] Blocher J., Eckert I., Elster J., Wiefek J., Eiffert H., Schmidt H. (2011). Aquaporins AQP1 and AQP4 in the cerebrospinal fluid of bacterial meningitis patients. *Neuroscience Letters*.

[27] Castañeyra-Ruiz L., González-Marrero I., Castañeyra-Ruiz M., González-Toledo J. M., Carmona-Calero E. M. (2014). Los canales de agua. Acuaporinas 1 y 4 en el sistema nervioso central y su relación con la hidrocefalia. *Majorensis*.

